# Improving the Referral Process, Timeliness, Effectiveness, and Equity of Access to Specialist Medical Services Through Electronic Consultation: Pilot Study

**DOI:** 10.2196/13354

**Published:** 2019-07-10

**Authors:** Véronique Nabelsi, Annabelle Lévesque-Chouinard, Clare Liddy, Maxine Dumas Pilon

**Affiliations:** 1 Département des Sciences Administratives Université du Québec en Outaouais Gatineau, QC Canada; 2 Groupe de Médecine de Famille Universitaires de Gatineau du Centre Intégré de Santé et des Services Sociaux de l’Outaouais Gatineau, QC Canada; 3 Department of Family Medicine University of Ottawa Ottawa, Ontario, ON Canada; 4 CT Lamont Primary Health Care Research Centre Bruyère Research Institute Ottawa, ON Canada; 5 Department of Family Medicine McGill University Montréal, QC Canada; 6 Collège Québécois des Médecins de Famille Montréal, QC Canada

**Keywords:** primary health care, referrals, physicians, specialists, health information systems

## Abstract

**Background:**

Access to specialty care remains a major challenge in the Canadian health care system. Electronic consultation (eConsult) services allow primary care providers to seek specialist advice often without needing the patient to go for a face-to-face consultation. It improves overall access to specialists and the referral process using an electronic care consultation service in urban and rural primary care clinics. This study describes the preliminary results of a pilot study with an eConsult service across 3 regions in the province of Quebec, Canada.

**Objective:**

The main objective of this study was to provide a 1-year snapshot of the implementation of the eConsult Quebec Service in rural and urban primary care clinics to improve access to care and the specialty referral process for primary care providers (PCPs).

**Methods:**

We established an eConsult service that covers urban and rural communities in 3 regions of Quebec. We conducted a quantitative analysis of all eConsult cases submitted from July 4, 2017, to December 8, 2018.

**Results:**

For over a year, 1016 eConsults have been generated during the course of this study. A total of 97 PCPs submitted requests to 22 specialty groups and were answered by 40 different specialists. The most popular specialty was internal medicine (224/1016, 22%). Overall, 63% (640/1016) of completed cases did not require a face-to-face visit. PCPs rated the service as being of high or very high value for themselves in 98% (996/1016) of cases.

**Conclusions:**

The preliminary data highlight the success of the implementation of the eConsult Quebec Service across 6 primary care clinics. The eConsult platform proves to be effective, efficient, and well received by both patients and physicians. If used more widely, eConsult could help reducing wait times significantly. Recently, the Ministry of Health and Social Services of Quebec has identified developing a strategic plan to scale eConsults throughout other regions of the province as a top priority.

## Introduction

### Background

Wait times for specialized medical care are a major issue in the Canadian health care system [1-4]. Patients wait several weeks or months for an initial appointment. The Commonwealth Fund’s 2016 enquiry, conducted in 11 industrialized countries on timeliness of care, has ranked Canada last in terms of wait times for specialized health care [5]. A large percentage of Canadian family physicians reported long waits for specialist consultation and procedures [6]. The average wait time to see a specialist after referral from a primary care provider (PCP) increased substantially from 3.7 weeks in 1993 to 9.4 weeks in 2016 [7]. At the provincial level, Quebec is faced with the same challenges as the other provinces. The impact of waiting for access to specialists is significant for patients, with longer delays increasing stress, anxiety, and pain, affecting daily activities and sometimes leading to deterioration in health [2]. Delayed access to specialist care can result in diagnostic delays, duplication of tests and services, dissatisfaction among patients and providers, and rising costs [2,8].

### Deficiencies in the Current Referral Process

The referral process from primary care to specialty care has several weaknesses that can lead to a duplication of tests, multiple medications taken for the same health condition, and deterioration of patient health [9,10]. However, the issue of wait time extends beyond the amount of resources available; an investigation of the causes of wait times is necessary [11,12]. Problems at various stages of the specialist referral process are mentioned in the literature. An incomplete gathering of patient information [9,13-16] and inadequate screening [13,17,18] are 2 initial shortcomings that may be encountered. The knowledge and expertise of the PCPs as well as their work environment are important factors that may influence triage [19] and presence or absence of referral [9]. Referral to the wrong specialists may also occur [20].

Second, the quality of the referral is often lacking [21]. A Canadian study that surveyed 3000 general practitioners and specialists showed that 51% of the referrals were inadequate in that the reason for referral was unclear [22]. Moreover, when the referral is lacking essential patient information [9], the specialist must then collect this information, increasing wait times and likely delaying clinical decisions. Gandhi et al [16] noted in a study comprising 48 general practitioners and 200 specialists that the primary source of delay was in the gathering of patient data. Clearly, inefficient communication is a source of ambiguity or confusion for specialists [9].

Mehrotra et al [9] also noted ambiguity with regards to coordination of patient care. There may be miscommunications concerning who will take responsibility for patient care as well as disagreements upon the treatment plan. Patients may be left with contradicting information. Stille et al [15] observed that parents of 38% of pediatric patients were required to transmit information from 1 physician to another and that most parents were uncomfortable fulfilling this role. It seems up to 50% of new appointments with some categories of specialists are individual patients referring themselves. Patient dissatisfaction with the traditional referral model may be 1 explanation for this phenomenon [23]. The literature review highlights multiple issues with our current referral process [15,16,24,25]. As the Haggerty et al [26] continuity of care model shows, issues in the referral process may arise on an informational, administrative, or relational level. We believe that there is a need to improve the quality of the communication and collaboration between PCPs and specialists to optimize patient care and safety.

### Process-Based Solution

On the basis of a systematic review, 1 of the interventions highlighted by Blank et al [17] is aimed directly at the referral process. These include interventions such as insuring communication between the general practitioner and the specialist before referring and electronic systems for referral as well as support for decision making.

Liddy et al have been tackling these issues by developing, implementing, and evaluating an innovative electronic health solution called the Champlain BASE (Building Access to Specialists through eConsultation) service [2,3,27,28]. This eConsult service has been extensively tested in eastern Ontario region, Canada, and currently operates as a fully funded program. eConsult BASE innovation has been reported to improve coordination in health systems by allowing direct communication between PCPs and specialists, improving access to shared records, and improving continuity of care by providing direct access to multiple specialty types [1,2,27,28]. eConsult BASE services reduce wait times for specialists, avoid unnecessary referrals, and therefore, have a large impact on costs [24,29]. Finally, PCPs, specialists, and patients were highly satisfied [29].

Liddy et al [27] found that the family physicians using the service feel more confident when treating their patients. They also appreciate the educational aspect of this platform, which allows them to better manage certain medical conditions in the clinic. Keely et al [2] report that the specialists using this platform say it allows them to be more innovative in patient care and improves their communication with family physicians. In addition, Keely et al [29] demonstrated that 50% of the electronic consultations (eConsults) conducted in endocrinology, hematology, and dermatology were answered without the patient and the specialist needing to meet in person, as it normally would have taken place. However, it is reported that patients have mixed views concerning this electronic platform. A total of 46% of patients believe that it presents a viable alternative to face-to-face meetings with the specialist, as it reduces time and effort to set up a meeting with a specialist and allows them to get answers more rapidly [30]. Johansson et al [31] reported that video consultation would facilitate access to health specialists for those living in rural areas and for the elderly. This being said, 46% of the patients interviewed said they were uncertain if they preferred a video consultation over an in-person encounter.

The College of Family Physicians Canada shared these results with their provincial section. Among them, the Quebec College of Family Physicians (QCFP) chose to tackle the project of implementing an eConsult-like service within the province. A team was gathered in 2016 bringing together the QCFP, RUIS McGill Telehealth, Centre intégré de santé et de services sociaux de l’Outaouais, Centre intégré universitaire de santé et de services sociaux de la Mauricie-et-Centre-du-Québec, and Centre intégré de santé et de services sociaux de l’Abitibi-Témiscamingue with the mentorship of the Champlain BASE eConsult team. The team adopted a governance that ensured the coordination of the activities under the entity of eConsult Quebec. In 2017, the team joined the pan Canadian Connected medicine initiative from the Canadian Foundation for Healthcare Improvement, which was a learning collaborative intended of supporting the spread and scale of eConsult BASE.

The objective of this study was to describe the initial experience with the implementation of eConsult Quebec Service in rural and urban primary care clinics.

## Methods

### Study Setting

**Figure 1:**
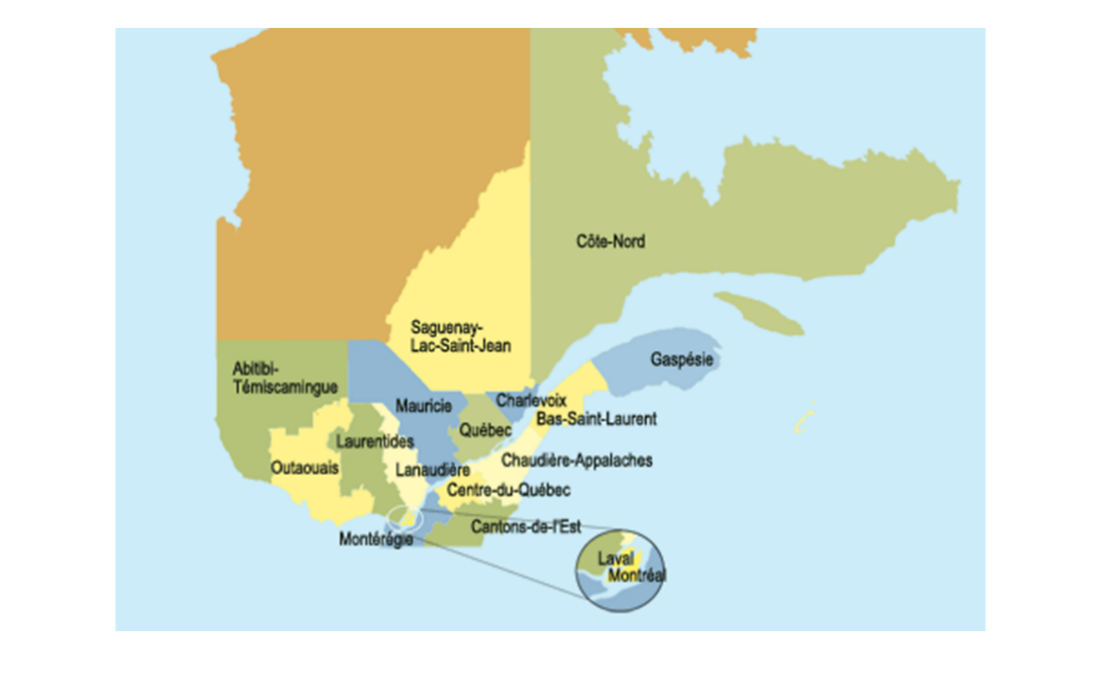
Map of Quebec.

This study took place in 6 clinics (4 urban and 2 rural) located across 3 different regions of the province of Quebec: Outaouais, Abitibi-Témiscamingue, and Mauricie. Quebec is the largest of Canada’s 10 provinces in area and is second only to Ontario in population. Figure 1 shows the extent of the 3 regions. The current population of Outaouais is 382,604 and has a land area of 30,504 km^2^. As Outaouais, Abitibi-Témiscamingue is located in western Quebec, Canada, with a population of 145,690 and a land area of 57,726 km^2^. Mauricie has the largest population with 512,300 and has a land area of 45,000 km^2^.

All 3 regions volunteered to be early adopters of the service and identified significant access issues and delays for specialists’ appointments in their regions.

The first phase, in the Outaouais region, involved 2 primary care clinics (1 rural and 1 urban), which included 25 specialists representing 20 specialties and 29 PCPs. This first phase began in July 2017. The second phase, which began in February 2018, corresponded to the deployment of eConsult service in the Abitibi-Témiscamingue region. This phase included 3 primary care clinics (2 urban and 1 rural), with 10 specialists representing 7 specialties and 41 PCPs. The third phase, which began in April 2018, was held in the Mauricie region. A total of 1 urban primary care clinic was involved, with 5 specialists representing 5 specialties and 27 PCPs. To meet the needs of PCPs, some specialists have agreed to respond to the requests from all of the regions.

Study participants have been recruited from urban and rural primary care practices across the 3 regions of Quebec. From the very beginning of this initiative, a few PCPs and specialists proposed themselves to be a champion to enrolled participants but also PCPs were self-identified after learning about the service through presentations or word of mouth. Specialty services were added based on feedback from the primary care participants and interest expressed from specialists.

#### Development of the Electronic Consultation Service

An eConsult service was established in 2010 by author CL and Dr E Keely in partnership with the Champlain Local Health Integration Network, The Bruyère Research Institute, and Winchester District Memorial Hospital. CL and her Champlain BASE project team were attempting to respond to the challenges of referrals between PCPs and specialists by developing, implementing, and evaluating a secure online platform for eConsult. Given the success of the eConsult concept in Ontario and other regions of Canada, the leadership as described above, developed and implemented an eConsult service tested in 3 regions of the province of Quebec. There are variations in the design of the eConsult service because Quebec is different from the other jurisdictions in Canada on a number of fronts, including policy and regulations (eg, licensing, privacy, and liability), financing (eg, provider remuneration) and, of course, language, where French is the majority language as opposed to English elsewhere.

The Champlain BASE business model was replicated onto an enterprise telehealth platform already in operation on the Quebec Healthcare Network. Privacy impact and threat risk assessments were also performed in compliance with the Personal Health Information Protection Act of the Ministry of Health and Social Services of Quebec.

#### Design of the Service Workflow

Like the Champlain BASE eConsult service, eConsult Quebec is a service providing a platform for communication between PCPs and specialists. It is a secure Web-based application that allows PCPs to submit questions to specialists, to gain insight on the best management plan for patients.

The eConsult begins with a PCP’s clinical question. The PCP submits their question via a standardized secure Web form, along with any relevant demographic information and supplementary files (eg, photos, lab results, electronic medical record–generated letter, and pictures of cutaneous lesions). The form is kept extremely simple and focused to ensure favorable user adoption. A centralized coordinator receives all PCP requests and dispatches them to participating physicians of the appropriate specialty. The specialist receives an email prompt, is given 7 days to respond to the request, and is remunerated at a rate of Can $200 per hour prorated to their self-reported time required to complete the eConsult. For every consult, the specialist offers clinical recommendations, may ask for further clarification from the PCP, or may suggest an in-person consultation. PCPs and specialists may correspond back and forth until the PCP closes the request.

**Figure 2:**
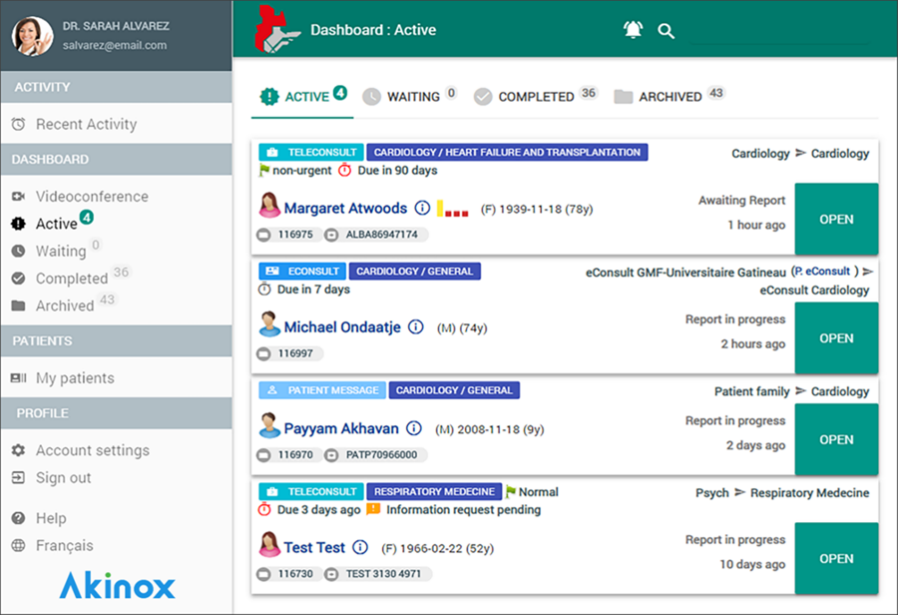
Dashboard of the eConsult (electronic consultation) Quebec Service.

The user’s dashboard (Figure 2) contains all requests across all communication types (eConsults, patient forms and messages, teleconsultations, and transfer requests). The view filters and possible actions are dynamically adapted according to user roles and permissions. Patient data are encrypted and accessible only to the physicians involved in the eConsult or their delegated staff through role-based access control. The platform is installed on-premise in a secure data center within Quebec’s official dedicated health network, complies with all applicable governmental regulations, and is audited weekly for security (vulnerability assessments etc).

User may choose card view (as shown in Figure 2) or table view. Main common status filters are as follows:

- Active: contains all pending actions for the current user groups. Draft eConsults and those requiring a response or further tasks to complete are shown here.
- Waiting: requests awaiting actions from others, such as a specialist’s response.
- Completed: after all actions are completed, item is shown here for reference.
- Archived: optionally, users may choose to archive completed items, in which case they will be shown here. Organizations may choose to implement automatic archival parameters (not the case for this project at present). An archived case remains fully accessible.

In addition, we have defined the role of each user group that control access to the platform for easy management of security (Table 1).

In preparation for eConsult Quebec service implementation, test and demonstration environments separate from the production environment were used by PCPs and specialists to evaluate the platform, suggest improvements, experiment, train, and support change management efforts. A training group session was offered for all participants but was not mandatory, and training videos were available too.

We identified the specialties needed by region with “Wait Times 1” to identify the specialties that were the most referred to and with the longest wait times. Wait Times 1 is defined as period between the initiation of a referral by a PCP and the moment the patient sees that specialist. With the information provided by “Wait Times 1,” the local champions were asked to recruit the identified specialties per region. Ongoing communications with the PCP allowed us to identify new specialties that would add value to the services.

### Assessment

Following completion of the eConsult, the PCP receives a mandatory close-out survey to rate the outcome and the value of the interaction with a 5-question close-out survey (Textbox 1). Question 1 asks about the perceived usefulness of the advice the PCP received from the eConsult. Question 2 asks about the result of the eConsult in regard to referral. Questions 3 and 4 are answered on a 5-point Likert scale and ask PCPs about the value of the service for their patients (Q3) and themselves (Q4). The last is an open-ended question (optional) for any additional comments about the eConsult service (Q5).

#### Data Sources

We conducted a quantitative analysis of 1016 eConsult cases completed from July 4, 2017 to December 8, 2018. We used a combination of on-going real-time system utilization data collected through the eConsult Quebec service. Briefly, for each eConsult case submitted at all primary care clinics, the system automatically collects data regarding the PCP, the consulting specialist, the clinical questions posed, and the answers provided. The system also collects data on the user’s log-in time, time spent on the consultation, time for reply, closure of the case, and responses to a mandatory satisfaction survey completed after each eConsult case is closed.

This study was approved by the institutional review ethics board of the Integrated Health and Social Services of the Centre of Outaouais.

### Ethics Approval

Ethical approval was obtained from the research ethics board of Centre intégré de santé et des services sociaux de l'Outaouais (ref. number 2016-183_88) in Quebec, Canada. This study did not include direction patient contact, and thus, formal consent was not obtained.

**Table 1:** User groups permissions and authentication.

User Groups	Description
Requesting clinician	Can create new electronic consultations (eConsults), follow-up on existing ones, complete the close-out research survey, and archive own completed eConsults (typically primary care practitioners: general practitioner, family physician, nurse).
Requesting clinician delegate	Able to accomplish all tasks of the requesting clinician but as a delegate. The system contains a full audit trail with history of changes, and all delegated actions are shown as (Delegate Name) on behalf of (Clinician Name; typically primary care practitioners: general practitioner, family physician, nurse).
Responding clinician	Responds to eConsults, can request further information or documents, specify whether a referral is required, and archive own completed eConsults (typically specialists).
Responding clinician delegate	Able to accomplish all tasks of the responding clinician but as a delegate. The system contains a full audit trail with history of changes, and all delegated actions are shown as “(Delegate Name) on behalf of (Clinician Name)” (typically specialists).
Dispatch	Assigns incoming eConsults to specialists depending on availability, conditions, etc. Reviews the status dashboard to ensure process goes smoothly and eConsults are answered in a timely manner (typically planning, programming, and research officer).
Super dispatch	Same permissions as dispatch but views all cases regardless of group, institution, or network (typically planning, programming, and research officer).
Manager	Access to business intelligence dashboards, reports, and statistics.
Administrator	Manages users, groups, and other system parameters.

**Mandatory close-out survey completed by primary care providers at the end of each electronic consultation.**

Q1. Which of the following best describes the outcome of this electronic consultation (eConsult) for your patient:

1. I was able to confirm a course of action that I originally had in mind
2. I got good advice for a new or additional course of action
3. I did not find the response very useful
4. None of the above (please comment)

Q2. As a result of this eConsult, would you say that

1. Referral was originally contemplated but now avoided at this stage
2. Referral was originally contemplated and is still needed—this eConsult likely leads to a more effective visit
3. Referral was not originally contemplated and is still not needed—this eConsult provided useful feedback/information
4. Referral was not originally contemplated, but eConsult process resulted in a referral being initiated
5. There was no particular benefit to using eConsult for your patient in this case
6. Other (please comment)

Q3. Please rate the overall value of the eConsult service in this case for your patient:

Minimal 1 – 2 – 3 – 4 – 5 Excellent

Q4. Please rate the overall value of the eConsult service in this care for as primary care provider:

Minimal 1 – 2 – 3 – 4 – 5 Excellent

Q5. We would value any additional feedback you provide:

*[Optional free text field]*

## Results

### Assessment of the Electronic Consultation Quebec Service

**Figure 3:**
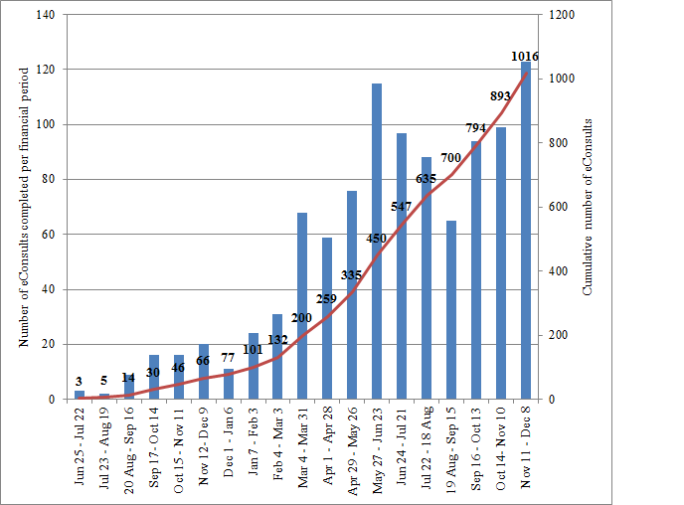
Electronic consultation (eConsult) case volume—the number of cases completed per financial period and cumulative total.

The eConsult service has shown a great interest during its implementation for the province of Quebec. A total of 97 PCPs (94%) of the 103 registered in 3 regions completed 1016 referrals during the 19 months. Figure 3 illustrates the eConsult case volume for all regions per financial period based on the calendar of the Ministry of Health and Social Services of Quebec. The first eConsult in the Outaouais region was July 1, 2017, and the first cases in Abitibi-Témiscamingue and Mauricie were January 16, 2018, and March 28, 2018, respectively. The monthly volume of cases started slowly in the last 2 quarters of 2017 but grew more rapidly after the first quarter of 2018 (see Figure 3).

The breadth of specialties accessed by patients is shown in Table 2. A total of 97 PCPs (94%) of the 103 registered submitted at least 1 eConsult. A total of 97 PCPs submitted requests to 22 specialty groups and answered by 40 different specialists. The most commonly referred to specialties were internal medicine (224/1016, 22%), dermatology (203/1016, 20%), gynecology/obstetrics (117/1016, 12%), endocrinology (75/1016, 7%), and orthopedics (57/1016, 6%), followed closely by psychiatry (50/1016, 5%) and gastroenterology (47/1016, 5%).

Specialists provided a response in an average of 4 days. In 87% of cases (884/1016), they took less than the 7-day response period. The self-reported time specialist spent completing the referral was 12.43 min.

The self-reported time it took for a specialist to complete the eConsult (specialties that had 13 or more completed cases; N=986) was less than 10 min in 29% of cases, 10 to 15 min in 54% of cases, 15 to 20 in 7% of cases, and over 20 min in 10% of cases (Table 3).

In 77.1% of cases (783/1016), PCPs who submitted a request required a single correspondence with the specialist and 19.4% (197/1016) required 2 correspondences to clarify or collect further information on the clinical case. The maximum number of correspondences was 6 for just 1 case (Table 4).

The most common question types were based on treatment, general management, investigation indications, diagnostic, test interpretation, prognostic, resource availability, and continuing education.

Adaptations to the Akinox Platform support the eConsult Quebec Service cost Can $25,000. The delivery costs of eConsult in 3 regions of Quebec were Can $31,395, which includes user setup and registration, user support, flow, operational support and hosting services, and administrative costs. The cost of remunerating specialists was Can $23,000. The total cost of the eConsult Quebec Service during the period study was Can $79,395.

Table 5 reports the average specialist remuneration cost per specialty (for specialty groups that had 13 or more completed cases; N=986). For 13 or more completed cases, the less expensive were cardiology (24 cases) for Can $27.75/eConsult and infectious diseases (13 cases) for Can $28.18/eConsult, and the higher were gastroenterology (47 cases) for Can $67.31 and psychiatry (50 cases) for Can $66.93/eConsult.

**Table 2:** Specialty distribution.

Specialty groups	Electronic consultation case volume	Percentage
Anesthesiology	1	0
Dental medicine	2	0
Pain medicine	2	0
Ophthalmology	4	0
Fertility	6	1
Allergology	8	1
Pulmonary diseases	7	1
Infectious diseases	13	1
Neurosurgery	17	2
Cardiology	24	2
Ear, nose, and throat	25	2
Rheumatology	26	3
General surgery	30	3
Neurology	41	4
General pediatrics	37	4
Psychiatry	50	5
Gastroenterology	47	5
Orthopedics	57	6
Endocrinology	75	7
Gynecology/obstetrics	117	12
Dermatology	203	20
Internal medicine	224	22

**Table 3:** Self-reported time it took nonfamily physician specialists to complete electronic consultations (13 or more completed cases).

Time to complete electronic consultation	Referrals, n (%)
<10 min	286 (29)
10-15 min	536 (54)
15-20 min	67 (7)
>20 min	97 (10)

**Table 4:** Number of correspondence and correspondences between the primary care provider and the specialist per case (N=1016).

Number of correspondence and correspondences	Cases, n (%)
1	783 (77.1)
2	197 (19.4)
3	28 (2.8)
4	7 (0.7)
5	0 (0.0)
6	1 (0.1)

**Table 5:** Average specialist remuneration cost per electronic consultation (eConsult) and average specialist self-reported time to complete an eConsult (for specialty services that had 13 or more eConsults completed).

Specialty groups	Average specialist remuneration cost per eConsult (Can $)	Average time to complete (min)	Cases completed (n)
Gastroenterology	67.31	20.21	47
Psychiatry	66.93	20.10	50
Rheumatology	60.84	18.27	26
Neurology	50.36	15.12	41
Dermatology	43.14	12.96	203
Ear, nose, and throat	41.29	12.40	25
General surgery	40.52	12.17	30
General pediatrics	40.50	12.16	37
Internal medicine	38.87	11.67	224
Neurosurgery	35.26	10.59	17
Gynecology/obstetrics	31.73	9.53	117
Orthopedics	30.38	9.12	57
Endocrinology	29.30	8.80	75
Infectious diseases	28.18	8.46	13
Cardiology	27.75	8.33	24

**Figure 4:**
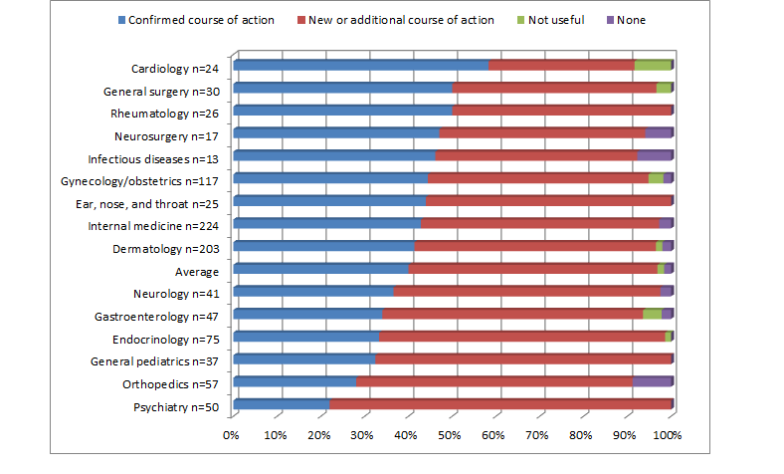
Impact of electronic consultation (eConsult) on the course of action by the primary care provider by specialty services that had 13 or more eConsults completed.

**Figure 5:**
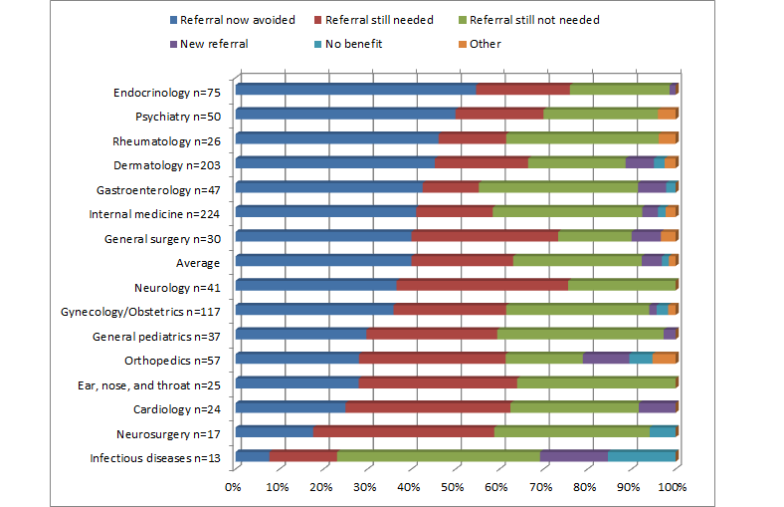
Impact of electronic consultation on need for face-to-face referral by specialty service.

During this study, 57% (563/986) of consults offered good advice for a new or additional course of action (see Figure 4). Merely less than 2% of cases were not found to be useful. A total of 97 PCPs (94%) of the 103 registered perceived 98% of eConsult cases to be of very good or excellent value for themselves. As illustrated in Figure 5, in 40% (394/986) of the cases submitted, a referral was originally contemplated but was now avoided. In 23% (227/986) of cases, a referral was originally contemplated and still needed—this eConsult likely leads a more effective visit. In 29% (286/986) of cases, a referral was not originally contemplated and was still not necessary, but the consultation allowed transmission of useful feedback or instruction. Overall, 63% (621/986) of completed cases did not require a face-to-face visit. These numbers varied across specialty services. In particular, endocrinology had the highest rate avoided referral with 52% (39/75), followed by psychiatry with 50% (25/50).

## Discussion

### Principal Findings

The results of our pilot study demonstrate that it is possible to spread an innovative model of care to improve access such as eConsult in another health system, and achieve similar uptake and outcomes as the original pilot program. The eConsult Quebec Service is the first eConsult service in the province of Quebec modeled after the Champlain BASE program. More than 1000 people received an eConsult during the study period and received advice within the expected parameters of the service except in rare cases. The service was established and offered multispecialty access across high-demand specialty areas. The costs were comparable with the implementation cost described in other jurisdictions [26,29]. The overall satisfaction of the PCPs was rated high, confirming the added value of this model in Quebec [29,32].

The eConsult BASE innovation has been reported as improving coordination within the health system by facilitating direct communication between PCPs and specialists, better access to shared records, and better continuity of care thanks to direct access to multiple providers across specialty areas [1,2]. eConsult BASE services reduce wait times for specialists, avoid unnecessary referrals, and have a large impact on cost savings through efficient care [27].

More recent data available of the experimentation of eConsult BASE in Ontario [33], among 33,327 eConsults, demonstrated that 67% of cases did not require a face-to-face consultation. Our study showed similar results with 63% of cases not requiring face-to-face consultation.

In Ontario, in 4% of cases, eConsult prompted a medical consultation, whereas this proportion reached 8% in Québec. The median response time was 0.9 days unlike 4 days in Québec [33]. One could think that this is most likely secondary to a volume effect. In fact, outliers specialists physicians with response time above the requested 7 days will affect more powerfully the average mean response time on a lower number of eConsults than on larger volume.

A surprising finding is the average time spent on an eConsult. Liddy et al [34] describe the 2 extremes of eConsults completed in less than 10 min and more than 20 min to be respectively 48.8% and 3.9%, whereas the Quebec experience finds these percentages to be 29% and 10%, suggesting that Quebec specialist are self-reporting longer times to answer eConsults. One could wonder about an experience effect; could specialists in Ontario have gained speed simply by using the service frequently and for a longer period of time? An alternative explanation could be cultural differences in corresponding: could Quebec specialist physicians write lengthier sentences, polite forms, and other variations explaining this discrepancy? An in-depth text analysis comparing both type of answers could clarify this question.

The specialty distribution of Quebec demonstrates a higher proportion of eConsult in internal medicine (22%) followed closely by dermatology (20%). This compares interestingly with BASE Champlain specialty distribution, with dermatology being the main specialty but not internal medicine, representing solely 3% of the total eConsults [35,36]. The high popularity of internal medicine is most likely explained by the limited availability of other specialties such as cardiology, hematology, and neurology in the all of the 3 regions. We expect these specialties to gain in interest as they become more available.

One new finding that this study brings is the number of iterations, showing that the majority of eConsults are resolved within 1 iteration; however, up to 20% of eConsult will require 2 iterations, explaining maybe the longer average response time observed.

Another interesting finding is the apparent correlation between the length of time spent on eConsult, the face-to-face consultations avoided, and the new or additional course of action section. Indeed, from the closing survey answer analysis, the top 5 specialties for the self-reported time to complete eConsult, (ie, gastroenterology, psychiatry, rheumatology, neurology, and dermatology) appear to be the same as the top 5 specialties for the highest proportion to “referral now avoided” as well as above the average for having a higher proportion of the category “New or additional course of action.” This may reflect knowledge gaps of PCPs in these specialties, creating an inverse correlation between the reflex of referring when knowledge is limited. To the contrary, cardiology has the shortest competition time, the highest “confirmed course of action,” and 1 of the top 3 lower percentage of “referral now avoided.” We expect that confirming an action takes less time than explaining “New a course of action.” Individual characteristics of physicians’ type may also explain these differences [37].

The above findings could help to inform continuous medical education as described by Archibald et al [38] and Davis et al [39]. This finding may also help guiding deployment strategies and remuneration discussions. This being said, the study was not designed to identify such an effect, and further research and perspective would be required to explore this topic thoroughly.

These findings confirmed that a Champlain BASE eConsult model could be replicated in Quebec with the same promising outcomes. Since completion of the pilot, the program continues to be offered and has received interim program–level support funding from the ministry of health. Plans are now underway to create a strategy to scale up the Quebec eConsult program across the whole population of 8. 39 million people.

Key implementation components included a focus on the local context of wait times, harnessing local clinical champions from primary and specialty care, engagement and commitment of local health service organization at both the individual clinic and regional level, and building on existing digital health assets to support the actual technology platform.

### Limitations

Although a strength of the study was the participation across 3 regions, this was on a voluntary basis, and thus, our very positive results may be affected by the selection bias of having particularly interested and motivated health care workers involved in the study. Our data were drawn from routinely collected utilization data. We did not directly interview patients nor collect patient level data to assess quality of actual eConsult response nor patient perspectives Future studies could be undertaken to explore barriers to the implementation of eConsult nationwide. We have collaborated with team members, a project manager, physicians’ champions, PCPs, specialists, and patient advisors to validate the organizational model, the processes, and the platform. These were tested in each primary care clinic, verifying that eConsult was adequately supported by local practices. This practical method of testing also allowed for participants to take ownership of the eConsult system, which secures future user uptake as participants have personally experienced the benefits of eConsult.

### Conclusions

Implementation of eConsult, a secure Web-based specialist consultation system, in Quebec was successful and resulted in overall similar outcomes that those observed in the Champlain BASE eConsult service. Some new insights about the correlations between eConsults self-reported time to completion and referral avoided warrant future research.

The eConsult Quebec Service is intended to replace, partially, traditional referrals from PCPs to specialists, thereby limiting wait times, patient inconvenience, and potential for miscommunication. Results from our eConsult project support its implementation in Quebec. The Ministry of Health and Social Services of Quebec made the scale-up of eConsult in primary health care 1 of their top priorities and are looking to put this innovation on the provincial policy agenda.

## Abbreviations

BASE: Building Access to Specialists through eConsultation
eConsult: electronic consultation
PCP: primary care provider
QCFP: Quebec College of Family Physicians
